# Supplementary material for: Determination of Fatty Acids Profile in Original Brown Cows Dairy Products and Relationship with Alpine Pasture Farming System
Source: Animals (Basel). 2020 Jul 20;10(7):1231. doi: 10.3390/ani10071231 (PMC7401626; doi:10.3390/ani10071231)
Supplement: Supplementary file 1 [file animals-10-01231-s001.pdf]

animals

Supplementary Materials: Determination of Fatty Acids Profile in Original Brown Cows Dairy Products and Relationship with Alpine Pasture Farming System

Stella Agradi [1,†], Giulio Curone [1,*,†], Daniele Negroni [1], Daniele Vigo [1], Gabriele Brecchia [1], Valerio Bronzo [1], Sara Panseri [2], Luca Maria Chiesa [2], Tanja Peric [3], Doina Danes [4] and Laura Menchetti [5]

[1] Department of Veterinary Medicine, University of Milan, Via dell'Università 6, 26900 Lodi, Italy; stella.agradi@studenti.unimi.it (S.A.); negroni.vet@gmail.com (D.N.); daniele.vigo@unimi.it (D.V.); gabriele.brecchia@unimi.it (G.B.); valerio.bronzo@unimi.it (V.B.)
[2] Department of Health, Animal Science and Food Safety "Carlo Cantoni", University of Milan, Via Celoria 10, 20133 Milan, Italy; sara.panseri@unimi.it (S.P.); luca.chiesa@unimi.it (L.M.C.)
[3] DI4A—Dipartimento di Scienze Agroalimentari Ambientali e Animali/Department of Agricultural, Food, Environmental and Animal Sciences, University of Udine, Via Sondrio 2/B, 33100 Udine, Italy; tanja.peric@uniud.it
[4] Facultaty of Veterinary Medicine, University of Agronomic Sciences and Veterinary Medicine, Splaiul Independentei 105, 050097 Bucharest, Romania; danes.doina@gmail.com
[5] Department of Veterinary Medicine, University of Perugia, Via San Costanzo 4, 06126 Perugia, Italy; laura.menchetti7@gmail.com
* Correspondence: giulio.curone@unimi.it; Tel.: +02503-17936
† These authors contributed equally to this work.

Table S1. Concentrate composition of both farms.

Cornflour, protein soybean meal, corn germ meal, dried sugar beet pulp, corn flakes, hulled sunflower seed meal, calcium carbonate (from ground limestone), soft wheat bran, corn gluten meal, soybean hulls, reed molasses, sodium bicarbonate, hydrogenates palm fat, sodium chloride, calcium diphosphate precipitate, magnesium oxide, dried yeasts from Kluyveromyces fragilis, Saccharomyces cerevisiae, Saccharomyces carlsbergiensis cultures	
Vitamin A	20000.00 UI
Vitamin D3	3250.00 UI
Niacin	162.50 mg
Vitamin E	125.00 mg
Zinc (zinc sulphate monohydrate)	100.00 mg
Manganese (manganese oxide)	75.00 mg
Iron (iron sulphate monohydrate)	45.30 mg
Copper (copper sulphate pentahydrate)	26.00 mg
Iodine (anhydrous calcium iodate, granules)	1.20 mg
Selenium (sodium selenite)	0.50 mg

Table S2. Correlation Matrix of fatty acids selected for Principal Component Analysis.

	C12:0	C14:0	C14:1 n9	C15:0	C16:0	C16:1 n9	C18:0	C18:1 n9c	C18:2 n6c	C18:3 n3	CLA n9c, n11t	C20:1 n9	C20:5 n3	C20:4 n6	C21:0
**C10:0**	0.688	0.436	0.418	-0.045	0.179	0.325	-0.374	-0.405	-0.120	-0.149	-0.370	-0.287	-0.131	-0.009	-0.170
**C12:0**		0.911	0.733	-0.249	0.682	0.306	-0.716	-0.616	-0.393	-0.522	-0.562	-0.344	-0.228	0.039	-0.332
**C14:0**			0.721	-0.181	0.866	0.114	-0.777	-0.678	-0.508	-0.531	-0.640	-0.260	-0.203	0.015	-0.359
**C14:1 n9**				0.035	0.705	0.354	-0.881	-0.484	-0.217	-0.181	-0.304	-0.110	-0.048	-0.138	-0.285
**C15:0**					-0.102	-0.090	-0.078	0.007	0.244	0.753	0.035	0.217	0.206	-0.277	0.226
**C16:0**						0.063	-0.823	-0.667	-0.596	-0.448	-0.537	-0.063	-0.130	0.047	-0.386
**C16:1 n9**							-0.197	0.239	0.232	-0.061	-0.151	-0.421	0.292	0.080	-0.104
**C18:0**								0.592	0.331	0.205	0.300	0.009	-0.068	0.055	0.144
**C18:1 n9c**									0.467	0.263	0.298	0.079	0.226	0.071	0.226
**C18:2 n6c**										0.577	0.303	-0.040	0.135	-0.281	0.145
**C18:3 n3**											0.362	0.280	0.382	-0.370	0.364
**CLA n9c, n11t**												0.327	0.307	-0.082	0.585
**C20:1 n9**													0.090	0.038	0.047
**C20:5 n3**														0.143	0.561
**C20:4 n6**															-0.071

Variables highlighted in grey were excluded from the PCA due to the high (> |0.90|) poor correlations (< |0.45|).

Table S3. Functions at group centroids. Unstandardized canonical discriminant functions evaluated at group means.

**Farm**	**Product**	
	**Milk**	**Cheese**
**Grazing system**	1.329	1.210
**No grazing system**	-1.329	-1.210
